# When Falls Reveal Acidosis: Unmasking Sodium-Glucose Co-transporter 2 (SGLT2) Inhibitor–Induced Euglycemic Diabetic Ketoacidosis

**DOI:** 10.7759/cureus.95681

**Published:** 2025-10-29

**Authors:** Japleen Kaur, Ashima Dogra, Anna Haymov

**Affiliations:** 1 Medicine, Government Medical College Amritsar, Amritsar, IND; 2 Hospital Medicine, Olean General Hospital, Olean, USA; 3 Department of Neurology and Neurosurgery, Lake Erie College of Osteopathic Medicine, Elmira, USA

**Keywords:** diabetes type 2, euglycemic diabetoketoacidosis, farxiga, high anion gap metabolic acidosis, sglt-2 inhibitor

## Abstract

Euglycemic diabetic ketoacidosis (euDKA) is a rare but serious complication of diabetes mellitus, consisting of about 3% of DKA presentations, and often associated with sodium-glucose co-transporter 2 (SGLT2) inhibitors. Unlike classic DKA, euDKA presents with ketoacidosis despite normal or only mildly elevated blood glucose levels, which can delay diagnosis and treatment. We report the case of a 77-year-old woman with insulin-dependent type 2 diabetes who presented after multiple unprovoked falls that appear to be multifactorial in origin. Initial evaluation revealed dehydration, mild hyperglycemia (glucose 183 mg/dL), elevated anion gap, and positive urine ketones and glucose. Despite treatment with intravenous (IV) fluids and subcutaneous insulin, her condition worsened over the next 24 hours, with progressive acidosis (bicarbonate <10 mmol/L, arterial pH <7.00) and glucose levels remaining below 250 mg/dL. EuDKA was diagnosed, but the patient was unstable and was moved to the ICU. Dapagliflozin was discontinued, and she was treated successfully with IV insulin and aggressive fluid resuscitation. This case illustrates the importance of maintaining a high index of suspicion for euDKA in elderly patients on SGLT2 inhibitors, particularly when presenting with nonspecific symptoms such as confusion or falls. Early recognition, prompt discontinuation of the SGLT2 inhibitor, and appropriate management are critical to improving outcomes.

## Introduction

Diabetic ketoacidosis (DKA) is a serious, potentially life-threatening complication of diabetes mellitus. It is traditionally characterized by markedly elevated blood glucose, anion gap metabolic acidosis, and the presence of ketones in the blood or urine. However, in a small subset of cases-approximately 2-3% of DKA presentations-patients may develop this condition without significant hyperglycemia, a phenomenon known as euglycemic diabetic ketoacidosis (euDKA), typically defined by a blood glucose level below 250 mg/dL [1]. This presentation can delay recognition and treatment. Older adults are particularly susceptible due to physiological changes that accompany aging, such as reduced thirst sensation, decreased kidney function, limited access to fluids or food, and the presence of multiple comorbidities [1].

The development of sodium-glucose co-transporter 2 (SGLT2) inhibitors, including dapagliflozin, canagliflozin, and empagliflozin, has transformed the management of type 2 diabetes mellitus. These medications not only help lower blood sugar but have also been shown to provide significant cardiovascular and kidney protection [2]. Despite these benefits, SGLT2 inhibitors have been linked to cases of euDKA, prompting a 2015 FDA safety warning to alert clinicians about this underrecognized adverse effect [3]. SGLT2 inhibitors lower blood sugar by causing the kidneys to excrete excess glucose in the urine. This process decreases circulating insulin levels while increasing glucagon, a hormone that promotes glucose and fat breakdown. During times of illness, dehydration, or poor food intake, this hormonal imbalance can shift metabolism toward fat breakdown and ketone production, leading to ketoacidosis without the high blood glucose levels typical of classic DKA [4,5]. Here, we describe a case of SGLT2 inhibitor-induced euglycemic DKA in a 77-year-old Caucasian woman who initially presented with recurrent falls. In her case, volume depletion and poor oral intake acted as the main precipitating factors.

## Case presentation

A 77-year-old Caucasian community living female with a history of insulin-dependent type 2 diabetes mellitus presented to the emergency department (ED) after being found lying on the floor by a neighbor with altered mental status. She had experienced multiple unprovoked falls over the prior three days, with the most recent fall occurring overnight. On further evaluation, she reported poor intake over the past week, particularly following an outpatient dental procedure. Her outpatient diabetes medication included insulin glargine 15 units SC daily, metformin 500 mg per os (PO) daily, dapagliflozin-propanediol (Farxiga^TM^) 10 mg PO daily, and liraglutide 1.8 mg PO daily.

**Figure 1:**
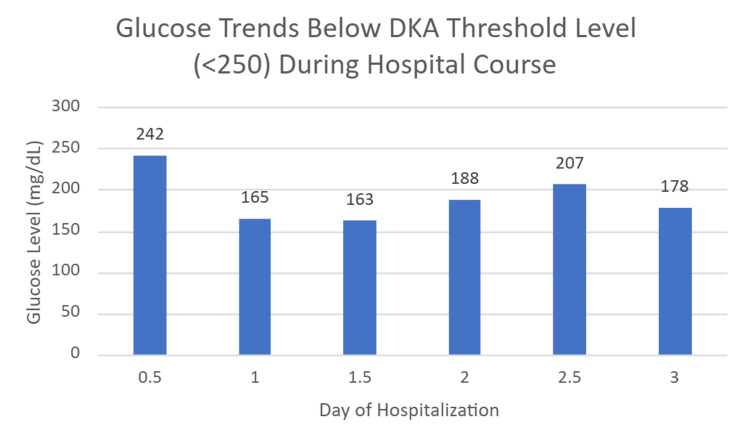
Glucose trending throughout hospital course The patient's glucose levels fluctuated throughout his admission but did not reach or exceed the value of 250 mg/dL, which is the diagnostic threshold for diabetic ketoacidosis.

On arrival at the ED, the patient was hemodynamically stable. Initial labs revealed an anion gap of 14.8, glucose of 242 mg/dl (Table 1, Figure 1), blood urea nitrogen (BUN) of 40 mg/dL, creatinine of 1.37 mg/dL, serum bicarbonate of 15.2 mmol/L, arterial base excess of -10.7, total creatine kinase of 299, and urine positive for ketones and glucose (Tables 1, 2).

**Table 1:** Basic metabolic panel Basic metabolic panel collected during the patient's first day of presentation.

Lab Parameter	Patient Value	Reference Value
Na	131	136-145 mEq/L
K	4.3	3.5-5.5 mEq/L
Cl	101	98-110 mEq/L
CO2	15.2	20-31 mmol/L
Anion Gap	14.8	4-13
Glucose	183	74-106 mg/dL
BUN	40	9-13 mg/dL
Creatinine	1.37	0.55-1.22 mg/dL
Creatine Kinase	299	34-145 U/L

**Table 2:** Urine analysis results HPF=High Power Field

Lab Parameter	Patient Value	Reference Value
Urine pH	5.5	4-8
Glucose (mmol/L)	>1000	Negative
Ketones (mg/dL)	40	Negative
Nitrite (mg/dL)	Negative	Negative
Leukocyte Esterase	Negative	Negative
WBC (WBC/HPF)	10	0-5
RBC (RBC/HPF)	5	0-3
Epithelial Cells (HPF)	>20	Few
Protein (g/L)	30	Negative

**Figure 2:**
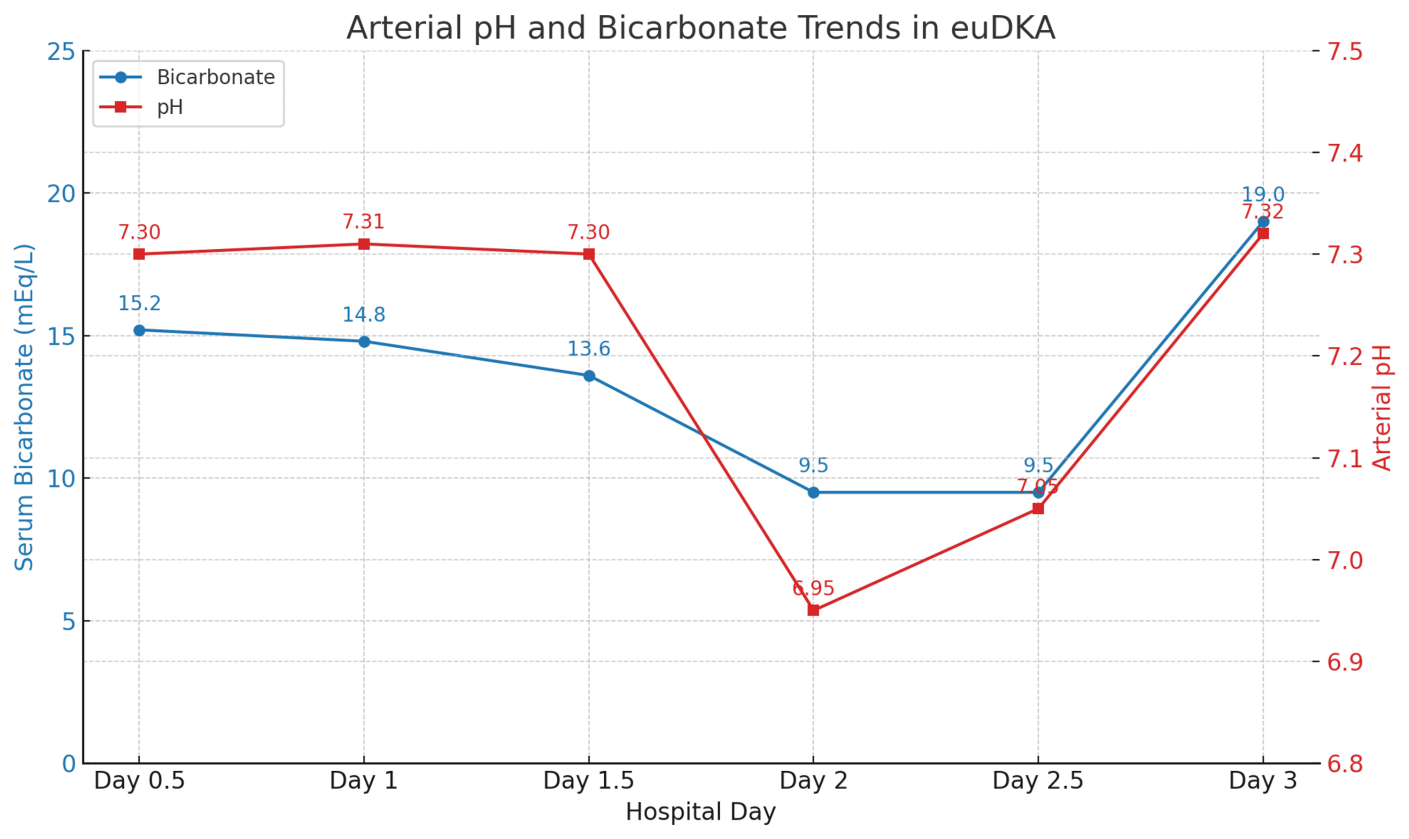
Arterial pH and bicarbonate trends throughout hospital course

There was no leukocytosis, and her chest X-ray and head CT were unremarkable. Pre-renal acute kidney injury (AKI) secondary to dehydration was suspected, which may further exacerbate ketogenesis. She was admitted to the inpatient medical floor. Her dapagliflozin and metformin were initially continued along with a sliding scale subcutaneous basal-bolus insulin regimen, and she was started on IV normal saline. The continuation of her medications at this point in her treatment, particularly the dapagliflozin, may have contributed to the metabolic worsening and delay in improvement of her symptoms. Over the next two days, the patient’s condition worsened. Clinically, she was confused and developed Kussmaul respirations characterized by deep and rapid breathing. Her anion gap rose to 21, serum bicarb fell to <10 mmol/L, and arterial pH dropped to <7.00 (Figure 2).

Two days after admission, her serum glucose was 188 mg/dL, which is below the threshold for typical diabetic ketoacidosis (Figure 1). Farxiga^TM^ was discontinued at this time. Her clinical and laboratory findings were consistent with euglycemic diabetic ketoacidosis (euDKA). She was transferred to the ICU, where she was managed with intravenous infusion of human regular insulin (100 mL total volume) at the rate of 4.3 mL/hour along with intravenous lactated Ringer’s solution. Over the next eight hours, she received around four liters of lactated Ringer’s. Her acid-base status gradually improved with arterial pH rising to 7.320 (Figure 2) and closure of the anion gap. Subsequently, she was shifted back to the floor and was discharged to subacute rehabilitation for physical therapy, as she had recurrent falls. Her SGLT2 inhibitors were temporarily discontinued, with a recommendation to follow up with her primary care provider to adjust her diabetic medications.

## Discussion

Sodium-glucose co-transporter 2 inhibitors (SGLT2i), such as dapagliflozin, are increasingly prescribed for the management of type 2 diabetes mellitus due to their proven efficacy in glycemic control and their cardiovascular and renal protective effects. However, a recognized but uncommon adverse event associated with this class of medications is euglycemic diabetic ketoacidosis (euDKA), a potentially life-threatening condition characterized by ketoacidosis without significant hyperglycemia. The risk of developing DKA in patients taking SGLT2 inhibitors is estimated to be up to 7-fold higher than in those not taking these agents [1,6].

This case highlights an atypical presentation of euDKA precipitated by fasting, dehydration, and continued SGLT2i use. The patient initially presented with multiple falls and was found to be dehydrated, with modest hyperglycemia, elevated anion gap, and ketonuria-findings that preceded the onset of overt DKA symptoms. Her progressive metabolic acidosis with a pH<7.3, bicarbonate level below 18, ketones in the urine, increased anion gap, and confusion, in the setting of normal to mildly elevated glucose levels, was ultimately consistent with the diagnostic profile of euDKA [5,6]. However, the nonspecific presentation with falls and altered mentation underscores the diagnostic challenges of euDKA, particularly in elderly patients.

The pathophysiology of SGLT2i-induced euDKA involves increased urinary glucose excretion, leading to decreased plasma glucose and insulin levels and a relative increase in glucagon secretion. This hormonal imbalance promotes lipolysis and hepatic ketogenesis. During periods of reduced oral intake, such as fasting, post-procedural recovery, or acute illness, insulin levels decline further, amplifying ketone body production. Because hyperglycemia is often absent or mild, recognition of this complication is frequently delayed, emphasizing the importance of clinician awareness [5,7,8]. In this case, the patient’s falls and confusion were the initial manifestations, rather than the classic triad of nausea, vomiting, and abdominal pain typically associated with DKA. Elderly patients, in particular, may exhibit blunted or nonspecific symptoms, making diagnosis even more difficult [2].

Initial management included intravenous fluids and subcutaneous insulin, but her SGLT2i therapy was inadvertently continued, which contributed to worsening ketoacidosis. Current clinical guidelines recommend holding SGLT2 inhibitors during acute illness, dehydration, or reduced oral intake to prevent DKA [9-14]. The FDA and multiple endocrine societies have issued warnings regarding this risk and emphasize adherence to “sick-day rules” for patients taking SGLT2 inhibitors. These recommendations include temporarily discontinuing the medication during periods of illness, dehydration, or fasting, maintaining adequate hydration and nutrition, and monitoring for signs of ketosis.

This case reinforces the need for a high index of suspicion for euDKA in patients on SGLT2 inhibitors who present with high anion gap metabolic acidosis, even in the absence of marked hyperglycemia. Prompt recognition, discontinuation of the offending agent, aggressive fluid resuscitation, and insulin therapy remain the cornerstones of management and are essential for achieving favorable outcomes [11,14].

## Conclusions

This case highlights the importance of maintaining high suspicion for euglycemic diabetic ketoacidosis in patients taking SGLT inhibitors, particularly in the setting of poor oral intake, dehydration, or nonspecific symptoms such as confusion or falls, particularly among the elderly. The absence of marked hyperglycemia may cause a delay in the diagnosis and management if clinicians are unaware of this presentation.

Early recognition, discontinuation of the offending agent, fluid resuscitation, and insulin therapy are critical for favorable outcomes. This case reinforces the need for patient education on the temporary cessation of SGLT2 inhibitors during periods of acute illness, fasting, or significant fluid loss, and for clinicians to monitor for euDKA even in atypical settings. Furthermore, interdisciplinary education, especially in geriatrics and primary care, where such presentations may be encountered, is extremely important to reduce incidences of missed diagnosis and subsequent failure to treat this condition. 
